# Establishment of an open-access canine breed-associated neurologic disease literature library

**DOI:** 10.4142/jvs.25316

**Published:** 2026-07-08

**Authors:** Kathryn Dalzell, Avinash Bevoor, Rebecca Wilson, Rodney S. Bagley

**Affiliations:** 1 College of Veterinary Medicine, Iowa State University, Ames, IA 50011, USA.; 2 Department of Veterinary Clinical Sciences, College of Veterinary Medicine, Iowa State University, Ames, IA 50011, USA.

**Keywords:** Dogs, breed-related, neurologic disease, library

## Abstract

**Importance:**

The Veterinary Neurologic Disease Library has been created as an educational resource library for veterinary professionals related to breed-associated neurologic diseases in dogs.

**Objective:**

To develop an open-access library that serves as a curated collection repository of peer-reviewed literature pertaining to dog breeds and neurologic diseases.

**Methods:**

Dog breeds were identified using the American and United Kennel Club registered breed lists. Disease terms were collected from published texts discussing veterinary neurologic diseases. Publicly available resources were searched by inputting keywords, including dog breeds, specific neurologic diseases, clinical signs, and additional related terms. Retrieved information was stored in a Zotero web-library, and automated searches continue to be conducted monthly. Original peer-reviewed manuscripts (in English) were categorized for inclusion with an individual unique breed was identified with a neurologic disease.

**Results:**

The library is currently active and accessible online. Four hundred breeds have been included for peer-reviewed citations of neurologic diseases. At the time of this writing, over 1,900 scholarly citations have been identified and categorized.

**Conclusions and Relevance:**

This centralized, open-access, repository of breed-associated neurologic diseases can be accessed to search breed and neurologic disease to assist primary care clinicians in the identification breed-associated neurologic disease in dogs and locating original source publications.

## INTRODUCTION

With hundreds of dog breeds recognized by the American and United Kennel Club (UKC), it has long been established that many disease processes have varying components of breed and genetic predilection [1]. Concurrently, as the scope of knowledge in veterinary medicine expands, the identification of unique genotypic and phenotypic disease characteristics is steadily increasing. As more knowledge is generated, this presents a challenge for clinicians to stay up to date with the continuously expanding list of diseases with known breed-associated predilections. In response, a repository of information on breed-associated neurologic diseases that can be easily accessed in a single location is a contemporary necessity for clinicians during clinical decision-making. Due to the complexity of disease pathophysiology expressed in an individual animal, however, the breed genotype relative may vary from the disease phenotype.

To facilitate access to scientific data related to breed-associated neurologic diseases, the Veterinary Neurologic Disease Library has been created [2,3]. This resource aims to offer veterinary professionals quick and efficient access to published data on breed-associated neurologic conditions via a centralized online library.

## METHODS

### Breed selection

A catalogue of individual dog breeds was generated using dog breed lists published on the American Kennel Club (AKC) and UKC web pages. Breeds with multiple recognized names (as determined by the AKC and UKC) were also catalogued and included in the search terms.

### Search process

The search process was initiated in January 2023 and performed monthly through the time of writing of this manuscript (Fig. 1). To identify a working list of neurologic diseases that have been reported in dogs, a constellation of neurologic diseases that have historically been recognized in specific breeds was compiled, as published in several textbooks on veterinary neurology [4,5,6,7]. Additional reported neurologic diseases were then identified utilizing the following online search engines: PubMed, Web of Science (Clarivate), Google Scholar, and Google search platforms. Keywords searched included combinations of “dog breed” and “neurologic disorder, disease” or “clinical signs” or additional related terms (Supplementary Table 1). The search was formulated to identify breed-associated neurologic diseases for inclusion in a curated collection and not as a component of a traditionally structured future systematic review. The Google search platforms were included as supplementary to the primary indexed databases.

**Fig. 1:**
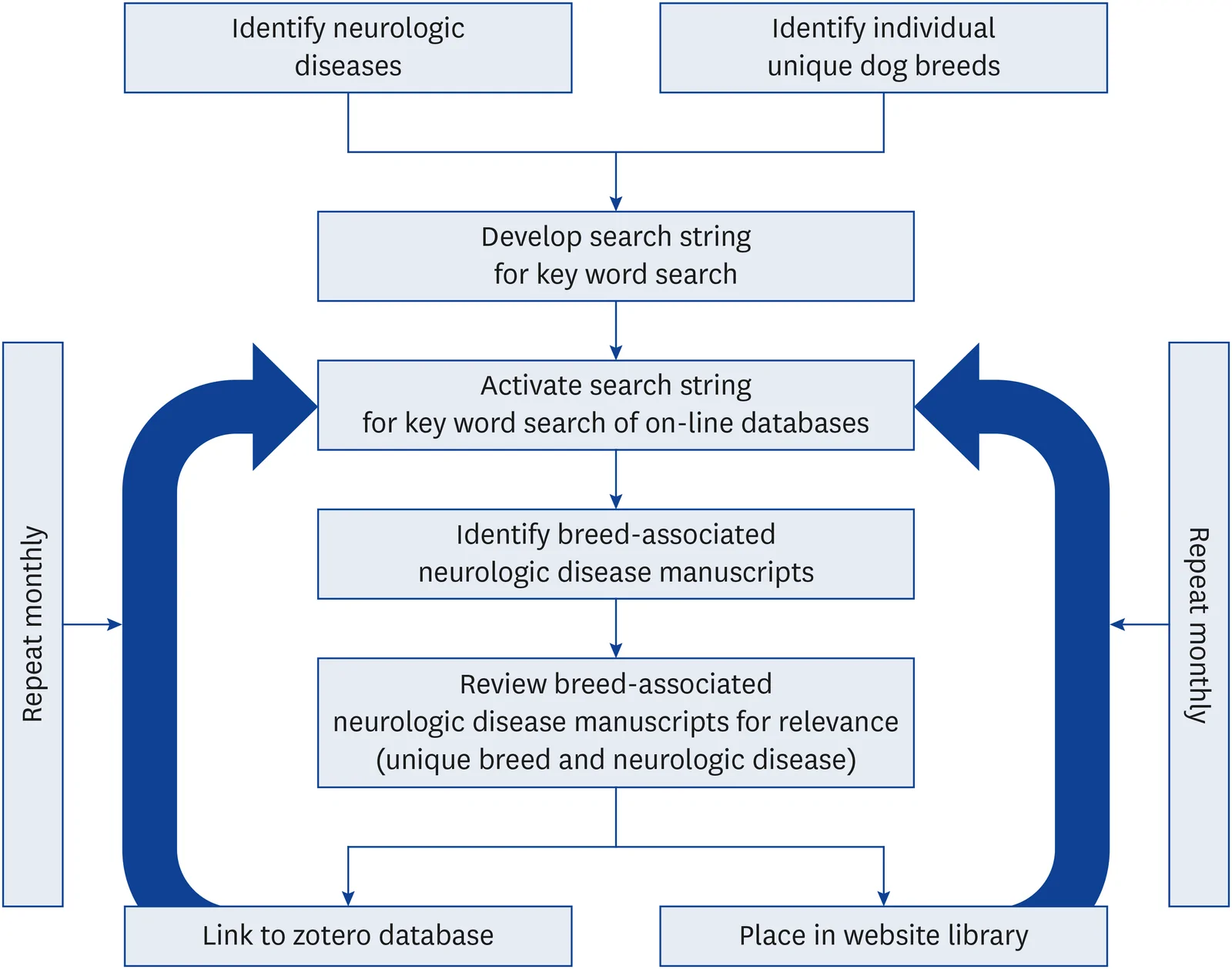
Workflow for creation of the Breed-related Neurologic Disease Library.

After this initial search, all keywords were compiled into a search string formed by two populations of terms; searched as “breed” AND “disease” (Supplementary Table 2) [8,9,10,11]. The “breed” component of the search string included all the dog breeds (and alternative breed nomenclature) listed in the AKC and UKC databases. The “disease” component of the search string contained all disease names and aliases previously identified. Within the search string, asterisks were applied at the end of a root word to expand search results to include variations of that root word. As an example, inputting “Spaniel*” in the search terms would populate articles mentioning “Spaniel” or “Spaniels.” In addition, including a term common to multiple breeds, such as “spaniel*” would produce search results that list any breed with “spaniel” in the name (such as Brittany Spaniel, English Springer Spaniel, or Cavalier King Charles Spaniel). This process was repeated for several other dog breeds listed (Supplementary Table 3).

We included peer-reviewed publications written in the English language describing naturally occurring neurologic diseases in dogs with stated breed associations. We excluded information from conference proceedings, theses, newsletters, and non-peer-reviewed web content.

Records were deduplicated in Zotero (current Version 8.0.3; Corporation for Digital Scholarship, accessed beginning January 2023) by DOI, title, and author/year. When the search string identified a manuscript containing the key words in either the title or the abstract, an initial review of the complete manuscript was performed by one of authors (KD, AB, RW) and subsequently reviewed by a board-certified veterinary neurologist (RSB) to ensure that the article identified includes an individual unique dog breed (not, for example, a “mixed” breed) and that the article included discussion of a neurologic condition. Disagreements were resolved by consensus. During the review no assessment was made of quality of evidence in each manuscript but rather only the correlation of breed and neurologic disease. Duplicates of the same manuscript were found in various search platforms were categorized as a similar reference. References managed in Zotero.

### Material collection and storage

Individual peer-reviewed references identified through the search process were added to the Zotero web-library [3]; organized by each breed and associated neurologic diseases. Publication categories included primarily case reports, case series, and genetic studies. Data extracted from each publication for the back-end library (not yet fully displayed on the public site) included breed, number of animals affected, sex, age of onset of signs, clinical signs, and other pertinent clinical information. This data was extracted into a standardized form including breed, sex, sample size, age of onset of clinical signs, clinical signs, clinical disease course, and genetic information (if available).

### Website development

The VNDL website was created using Weebly Free Website Builder Weebly (current version 5.60.1; Block Inc. Accessed December 2024). References are managed with Zotero web-library (version 8.0.3; Corporation for Digital Scholarship, accessed January 2023). A Zotero library containing all citations used to generate the database is available at the end of each disease page.

### Current updates to the primary database

Automated searches through Web of Science actively occur on the first day of every month by the authors. On the first day of each calendar month search results are manually reviewed. Peer-reviewed articles that emphasize phenotypic and/or genotypic descriptions of breed-associated neurologic diseases are downloaded for additional review as stated previously. If a new breed or disease term is identified, the newly identified term is searched as an individual combination as [(new breed) AND (diseases)] or [(all breeds) AND (new disease)] through Web of Science. The search string is then adjusted to ensure that all future searches will encapsulate references containing the newly identified term. Publications identified with each automated search are then updated to the Zotero web-library allowing new citations to be instantly available to those accessing the library. Furthermore, as new diseases and/or breeds are identified, they are added, in real-time, to the website, establishing this online resource as a historical repository with the potential to be updated in continuity with advancements in veterinary medicine and neurology.

## RESULTS

At the time of this writing, four hundred canine breeds recognized by the AKC and UKC are included in the search with associated neurologic disease. Across all these breeds, at present, 1,935 articles pertaining to breed-associated neurologic diseases have been identified and categorized. This curated collection of primary publications and review articles spans 89 years of published information regarding neurologic disease in dogs (1936 to 2025). The earliest date was based on the ability to view the original source data. Of the breeds evaluated, 273 of the 400 (68.3%) breeds had at least one associated neurologic disease in the primary literature. This website library is actively accessible using the links provided [2,3].

## DISCUSSION

Historically, medical professionals have relied on published materials and their individual memory of breed-associated neurologic diseases during assessment of patients with potential neurologic conditions. In the modern era, the expanding volume of clinical information presents a challenge for clinicians to utilize said information in a clinically expeditious manner. With the progression of technology and publication mechanisms, the contemporary approach now focuses on the ability of clinicians to understand how and where to search for clinical information in a readily accessible forum on an on-line platform. This library is collection of dispersed reference materials for rapid retrieval on breed-related neurologic diseases. Importantly, this library is a descriptive collection and does not purport to include or represent an analysis of the validity of individual source data.

In a clinical setting, when a veterinarian accesses the VNDL website, selecting the breed of the patient will bring the user to a collection of neurologic diseases specifically reported for, and associated with, that breed, which have been published in peer-reviewed scientific literature (English only). Selecting any disease will provide a resource link that directs the user to the Zotero web-library with manuscripts pertaining to that disease in that breed. This latter application provides the user with a list of all citations and the ability to gain access to the original reference for further information through open access or subscription-based mechanisms.

To expand this resource as a continuously evolving and “living” repository, further phases of development include the integration of extracted detailed descriptions of each disease, including the age of onset, sex predilection, clinical course, clinical signs, neuroanatomical localization, and genetic factors identified from the back-end library not currently available for public viewing.

While the goal of this repository is a comprehensive curated collection of published materials on breed-related neurologic diseases, there are known limitations regarding the breadth of collection at any time point. First, the search string formulated currently identifies articles that have a combination of the key terms mentioned in the title, citation, abstract, and/or tagged information related to the article. If these terms are not included in those sections of an article, the citation may not be immediately identified within the search results. Additionally, inconsistencies in disease nomenclature have created a challenge in collating certain diseases together as a single disease entity. In response, as each collected manuscript is found, any relevant citations mentioned in the primary article are cross-referenced with the Zotero database, and additional publications are added as they are identified. Another potential limitation of this database is that currently only articles available in the English language have been reviewed and categorized due to this being the primary language of the curators of this library. With the use of translational services and collaboration with international veterinary professionals, this library has the potential to expand to include scientific literature published in languages other than English.

Second, given that the core breeds used in this library we derived from the AKC and UKC recognized breed lists, there is a possible registry-based selection bias in this library. However, now that a core repository is available, future inclusion of a global spectrum of dog breeds recognized by additional kennel clubs is possible.

Third, categorizations of disease descriptions and terminology spanning decades present an inherent challenge. Due to the advancement of imaging modalities and genetic sequencing, as well as expansion of general knowledge within the field, disease classifications have been modified over time. As one example, primary reticulosis evolved to granulomatous meningoencephalitis in subsequent publications [12,13]. Accordingly, the curators of this database have had to interpret historical disease descriptions and place these within the modern context of what is currently understood about each disease process. As a result, information from references describing equivalent disease names was used when generating the general collection.

For this first iteration of curation, we have chosen to include only that information available in peer-reviewed scientific publications as the assurance of formerly appraised and peer-reviewed evaluation has been performed. In creating this library our intention was not to produce a traditional systematic review nor validate the scientific content of each piece within the collection. Our goal, rather, was to produce an easily accessible repository of information for use by others in the field. Ultimately, as with all information, individuals should interpret information within this collection in the context of individual patient characteristics and additional contextual clinical evidence.

Finally, given the vast landscape of current knowledge and the speed at which new information is generated, it is accepted that this library will be an expandible collection to be further supplemented by new knowledge and observations. As such, this Veterinary Neurologic Disease Library provides a mechanism for all veterinary professionals collectively to contribute to and expand the scope of known information in this repository.

## SUPPLEMENTARY MATERIALS

Supplemental Table 1 Search engines and terms used in preliminary search for neurologic conditions reported in dogs

Supplemental Table 2 Example search string used for comprehensive search of breed and neurologic disease combinations input through Web of Science

Supplemental Table 3 Example breeds included in search results with the use of a general breed term
